# Natural Killer Cell Phenotype and Function as a Predictive Factor for Treatment Response to Neoadjuvant Therapy in Breast Cancer Patients

**DOI:** 10.3390/ijms27041634

**Published:** 2026-02-07

**Authors:** Cinthya Yareli Anguiano Serrato, Fabiola Solorzano-Ibarra, Ignacio Mariscal-Ramirez, Maria Iyali Torres-Bustamante, Sylvia Elena Totsuka-Sutto, Jorge Raúl Vázquez-Urrutia, Aldo Alcaraz-Wong, Betsabé Contreras-Haro, Pablo Cesar Ortiz-Lazareno

**Affiliations:** 1 Hospital General Regional N°2 El Marqués, Instituto Mexicano del Seguro Social, Querétaro 76246, Querétaro, Mexico; anguiano@oncologia.org.mx; 2 Departamento de Biología Molecular y Genómica, Instituto de Investigación en Enfermedades Crónico-Degenerativas, Centro Universitario de Ciencias de la Salud (CUCS), Universidad de Guadalajara, Guadalajara 44340, Jalisco, Mexico; fabiolasolorzanoibarra@gmail.com; 3 Unidad Médica de Alta Especialidad, Departamento de Oncología Médica, Hospital de Especialidades (UMAE, HE), Centro Médico Nacional de Occidente Ignacio García Téllez, IMSS, Guadalajara 44340, Jalisco, Mexico; ignacio.mariscal@imss.gob.mx; 4 Centro Universitario de Ciencias de la Salud (CUCS), Universidad de Guadalajara, Guadalajara 44340, Jalisco, Mexico; iyali@hotmail.com (M.I.T.-B.); sylvia.totsuka@academicos.udg.mx (S.E.T.-S.); 5 Department of Medicine, The Pennsylvania State University College of Medicine, The Pennsylvania State University, Hershey, PA 17033, USA; jvazquezurrutia@pennstatehealth.psu.edu; 6 Unidad Médica de Alta Especialidad, Departamento de Patología, Hospital de Especialidades (UMAE, HE), Centro Médico Nacional de Occidente Ignacio García Téllez, IMSS, Guadalajara 44340, Jalisco, Mexico; aldo.alcaraz@imss.gob.mx; 7 Unidad de Investigación Biomédica 02, UMAE, HE, Centro Médico Nacional de Occidente Ignacio García Téllez, Instituto Mexicano del Seguro Social, Guadalajara 44340, Jalisco, Mexico; 8 Departamento de Ciencias Biomédicas, Centro Universitario de Tonalá (CUT), Guadalajara 45425, Jalisco, Mexico; 9 División de Inmunología, Centro de Investigación Biomédica de Occidente, Instituto Mexicano del Seguro Social, Guadalajara 44340, Jalisco, Mexico

**Keywords:** NK cells, cytotoxicity, breast cancer, pCR, Non-pCR, neoadjuvant therapy, cytokines, immune checkpoints, PD-1, TIGIT, NKG2D, DNAM-1, TIGIT/DNAM-1 axis, high-dimensional flow cytometry

## Abstract

Neoadjuvant systemic therapy (NST) is standard for locally advanced breast cancer (BC), yet predictors of pathological complete response (pCR) remain elusive. While Natural Killer (NK) cells are vital for anti-tumor response, their specific receptor dynamics during NST are poorly defined. This study provides a high-dimensional characterization of the peripheral NK cell landscape and immune signatures associated with therapeutic success. This prospective cohort study included 34 BC patients and 35 healthy donors (HD). Clinical characteristics were collected, and peripheral blood NK cell subsets were evaluated. We utilized high-parameter flow cytometry and unsupervised clustering (UMAP) to longitudinally track NK cell phenotypes (NKG2D, DNAM-1, PD-1, TIGIT) pre- and post-NST. NK cell cytotoxicity was evaluated, and serum levels of related IL-17A (interleukin), IL-2, IL-4, IL-10, IL-6, TNF-α (tumor necrosis factor-alpha), Fas, sFasL, IFN-γ (interferon-gamma), and Granzyme A were analyzed. Patients exhibited distinct NK cell profiles according to the pathological response. Only 12 BC patients achieved pCR. These patients showed improved NK cell cytotoxicity and higher concentrations of IL-2, TNF-α, sFASL, and Granzyme B after treatment compared with Non-pCR patients. In contrast, in Non-pCR patients, the percentages of CD56^bright^ NK cells increased after neoadjuvant therapy, whereas the more cytotoxic CD56^dim^ NK cell population decreased. Additionally, NK cells from Non-pCR patients exhibited higher co-expression of inhibitory checkpoints (TIGIT and PD-1), indicating reduced NK cell function. Otherwise, pCR patients displayed a more favorable balance of activating receptors (NKG2D and DNAM-1), and a favorable shift in the TIGIT/DNAM-1 activating-to-inhibitory axis. This study highlights the potential role of NK cells in determining the response to neoadjuvant therapy in BC patients. Those who achieved pCR showed enhanced NK cell activity and higher expression of activating receptors. Moreover, NK cells from Non-pCR patients showed lower cytotoxicity and higher expression of inhibitory receptors. These results suggest that NK cell phenotype evaluation could serve as a biomarker of treatment response in patients with BC. They also showed that the TIGIT/DNAM-1 axis can be a critical determinant of pCR.

## 1. Introduction

Breast cancer (BC) remains the most prevalent malignancy and the leading cause of cancer-related mortality among women worldwide, with incidence rates continuing to rise globally [1]. It is characterized by heterogeneity, not only in molecular subtypes (ranging from hormone receptor-positive to HER2 (Human Epidermal Growth Factor Receptor 2) and triple-negative disease) but also in the composition of the tumor microenvironment (TME) [2]. The TME is a complex, dynamic cellular ecosystem comprising immune cells, fibroblasts, endothelial cells, and stromal elements, all of which interact to shape clinical presentation, disease progression, and success of therapeutic interventions. For patients with locally advanced BC, neoadjuvant systemic therapy (NST) has become the standard of care, extending beyond tumor downstaging to facilitate breast-conserving surgery [3,4]. Despite these clinical goals, pathological complete response (pCR) rates remain low in certain patient subsets, underscoring the urgent need for predictive biomarkers [5,6].

Beyond its direct cytotoxic effects, NST systemically modulates immune architecture. Recent studies have highlighted that TME can suppress innate immune effectors at both metabolic and immunological levels [7,8]. Specifically, chemotherapy can reshape the immune landscape by altering the composition and function of Natural Killer (NK) cells [9]. As key effectors of the innate immune system, NK cells exhibit well-established antitumoral properties through the release of perforin and granzymes [10]. However, molecular crosstalk within the TME often leads to “functional exhaustion,” in which NK cells lose their efficacy to kill cancer cells, including breast cancer cells [11,12]. Furthermore, TME-induced metabolic reprogramming, characterized by hypoxia and nutrient competition, has been shown to severely limit NK cell glycolytic capacity, thereby diminishing their lytic potential before therapy initiation [13,14].

NK cell function is governed by a sophisticated balance between activating and inhibitory signals [15]. Activating receptors, such as DNAM-1 (DNAX accessory molecule-1, CD226) and natural killer group 2, member D (NKG2D), are essential for recognizing tumor-associated ligands, including MICA/B (MHC class I chain-related proteins A and B) and CD155 [16,17]. Conversely, upregulation of inhibitory checkpoints, such as PD-1 (Programmed cell death protein-1) and TIGIT (cell immunoreceptor with immunoglobulin and ITIM domain), has been associated with immune exhaustion [18,19]. This receptor competition affects NK cell functionality [20]. The TIGIT/DNAM-1 axis exemplifies opposing receptor functions and highlights the modulation of NK cell-mediated cytotoxicity by differential receptor expression [21]. Furthermore, current molecular evidence suggests that while NKG2D and DNAM-1 are essential for recognizing stress-induced ligands—such as MICA/B and CD155—on malignant cells [22], the tumor microenvironment (TME) often drives a functional “imbalance” by upregulating TIGIT and PD-1, which leads to NK cell exhaustion and immune evasion [19,23].

Serum cytokines and cytotoxic effectors—including IL-2 (interleukin), IFN-γ (interferon-gamma), TNF-α (tumor necrosis factor-alpha), Granzyme B, Perforin, and Granulysin—can serve as a vital window into systemic immune responses in cancer patients, providing a direct readout of the functional state of NK cells. These cells are immediate cytotoxic cells and critical immunomodulators; pro-inflammatory cytokines such as IFN-γ and TNF-α indicate an active functional state, whereas increased IL-10 or IL-4 may indicate a shift toward a suppressed, protumorigenic environment [24]. Furthermore, measuring Fas/FasL and soluble cytotoxic granules provides a quantitative assessment of NK cell function and its capacity to induce apoptosis in malignant cells [22,25]. Evaluating these soluble markers in relation to the TIGIT/DNAM-1 receptor axis is crucial, as it enables determination of whether cells are phenotypically activated or exhausted [23,26].

While the general relevance of tumor-infiltrating NK (tNK) cells has been established, the longitudinal dynamics of these receptors during the transition from pre- to post-NST remain largely undefined in clinical cohorts. The activation–inhibition balance of NK cells in blood can serve as a robust, non-invasive alternative to traditional tissue biopsies, offering a real-time window into treatment outcomes without the limitations of serial tumor sampling [27,28].

Our study provides a high-dimensional characterization of the activation receptor and immunocheckpoint landscape in peripheral NK cells, using multiparametric immunophenotyping to describe these co-expression profiles. We hypothesize that peripheral immune profiles, specifically the TIGIT/DNAM-1 axis regulation patterns, cytokine profile, and cytotoxic activity, will provide insights into the functional status of NK cells before and after therapies in patients who do or do not respond to NTS.

## 2. Results

### 2.1. Patient Characteristics and Clinical Demographics

The study included 34 patients with breast cancer (BC) and 35 healthy donors (HD). At baseline, no significant differences were observed between groups regarding age (55.2 ± 14.1 vs. 50 ± 9.6, *p* = 0.1), BMI (Body Mass Index) (27.9 ± 6.1 vs. 29.7 ± 5.3, *p* = 0.3), sedentary lifestyle (76.2% vs. 68%, *p* = 0.7), menopausal status (33.3% vs. 48.6%, *p* = 0.1), breastfeeding history (81% vs. 64%, *p* = 0.3), or contraceptive use (83% vs. 87%, *p* = 0.6). All BC patients completed their prescribed treatment without dose reductions or suspensions.

Patients were further stratified by clinical response to NST: 12 patients (35.2%) achieved pCR, while 22 (64.8%) did not (Non-pCR). As shown in Table 1, no significant differences between the pCR and Non-pCR groups were observed regarding age, contraceptive use, or histological grade. However, the pCR group showed a higher prevalence of Triple Negative and HER2+++ subtypes compared to the Non-pCR group (*p* = 0.03).

### 2.2. NK Cell Subpopulations Change Following Neoadjuvant Therapy

**Figure 1:**
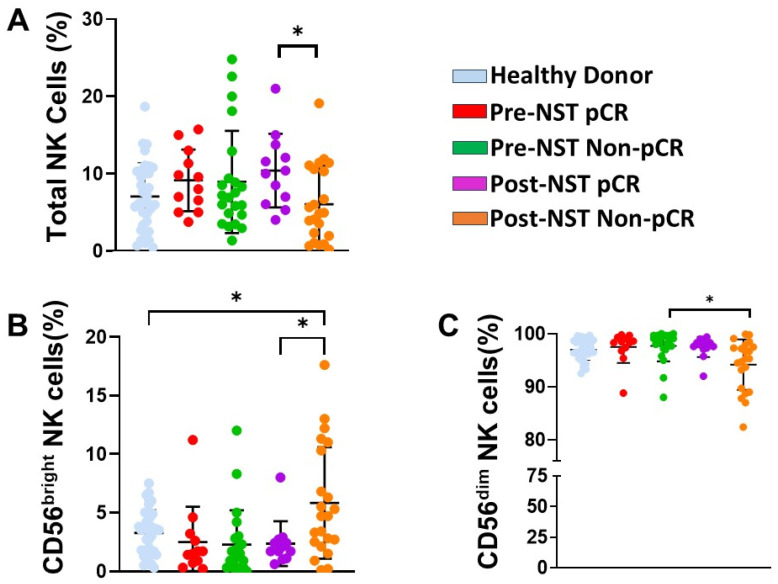
Natural Killer (NK) cell percentages were assessed in PBMC (peripheral blood mononuclear cells) samples from healthy donors (n = 35) and breast cancer patients (n = 34), stratified by pathological complete response (pCR) and non-pathological response (Non-pCR), both pre-neoadjuvant systemic therapy (pre-NST) and post-therapy (post-NST). We evaluated the percentages of peripheral blood: (**A**) total NK cells, (**B**) CD56^bright^ NK cells, and (**C**) CD56^dim^ NK cells from the different groups. The Y-axis represents the frequencies of the different cell populations, while the X-axis displays the study groups, each depicted in a distinct color. Data are presented as individual percentages of expression along with their mean values. Statistical significance among the five groups was assessed using a one-way ANOVA, followed by Dunnett’s multiple-comparison test. Only statistically significant comparisons are shown: * *p* ≤ 0.05.

We first examined whether NST altered peripheral blood NK cell percentages (Supplementary Figure S1). While total NK cell frequencies remained stable across the groups (Figure 1A), an exception was the Post-NST Non-pCR group, where we observed a reduction in NK cells in comparison with the Post-NST pCR group (*p* < 0.05; Figure 1A). CD56^bright^ NK cells significantly increased in Non-pCR patients following NST compared to both HD and pCR patients after NST (*p* < 0.05; Figure 1B), whereas CD56^dim^ NK cells significantly decreased in the post-NST Non-pCR group compared to the pre-NST Non-pCR group (*p* < 0.05; Figure 1C). The contraction of the cytotoxic CD56^dim^ NK cell pool and the expansion of the CD56^bright^ NK subset in Non-pCR patients after treatment (NST) indicate a failure to restore NK subsets.

### 2.3. Effect of NST on Peripheral NK Cell Populations Classified Based on the Expression of CD56 and CD16

To further characterize the impact of NST on NK cell populations with distinct maturation and effector potential, we subdivided peripheral NK cells into four different subsets based on the density of CD56 and CD16 surface expression: (A) CD56^bright^CD16^neg^ NK cells, (B) CD56^bright^CD16^pos^ NK cells, (C) CD56^dim^CD16^neg^ NK cells, and (D) CD56^dim^CD16^pos^ NK cells.

**Figure 2:**
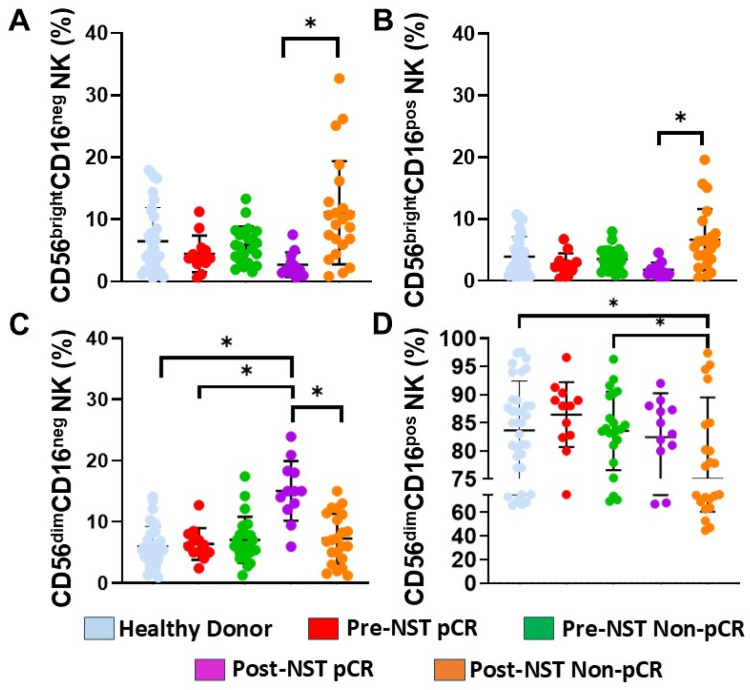
Percentages of NK cell subpopulations (based on CD56 and CD16 expression) were assessed in PBMC samples from healthy donors (n = 35) and breast cancer patients (n = 34), stratified by pathological complete response (pCR) and non-pathological response (Non-pCR), both pre- (pre-NST) and post- (post-NST) neoadjuvant systemic therapy. We evaluated the percentages of peripheral blood: (**A**) CD56^bright^CD16^neg^ NK cells; (**B**) CD56^bright^CD16^pos^ NK cells; (**C**) CD56^dim^CD16^neg^ NK cells; and (**D**) CD56^dim^CD16^pos^ NK cells from the different groups. The Y-axis represents the frequencies of the different cell populations, while the X-axis displays the study groups, each depicted in a distinct color. Data are presented as individual percentages of expression along with their mean values. Statistical significance among the five groups was assessed using a one-way ANOVA, followed by Dunnett’s multiple-comparison test. Only statistically significant comparisons are shown: * *p* ≤ 0.05.

Our analysis revealed significant phenotypic shifts associated with clinical response. In Non-pCR patients, a significant expansion of the immature CD56^bright^ CD16^neg^ population was observed post-NST compared to the post-NST pCR group (*p* < 0.05, Figure 2A). Similarly, the CD56^bright^CD16^pos^ subset—often considered a transitional maturation state—showed a significant increase in the post-NST Non-pCR patients compared with post-NST pCR patients (*p* < 0.05, Figure 2B)

In contrast, CD56^dim^CD16^neg^ NK cells significantly increased in the pCR group post-NST compared to HD, the pre-NST pCR group, and the post-NST Non-pCR group (*p* < 0.05, Figure 2C). Conversely, the most mature effector subset, CD56^dim^CD16^pos^ NK cells (Figure 2D), was significantly lower in the Non-pCR patients post-NST than in HD and in pre-NST Non-pCR patients (*p* < 0.05).

The accumulation of immature and transitional CD56^bright^ subsets, alongside the depletion of mature CD56^dim^CD16^pos^ effectors, in Non-pCR patients after treatment (NST) indicates profound impairment in NK cell maturation and recruitment following therapy. Conversely, the expansion of the CD56^dim^CD16^neg^ compartment in patients with pCR suggests successful mobilization of a cytotoxic pool, indicating the restoration of mature NK cell subsets after therapy.

### 2.4. UMAP Analysis Identifies a Distinct NK Cell Cluster in Healthy Donors Absent in Breast Cancer Patients

**Figure 3:**
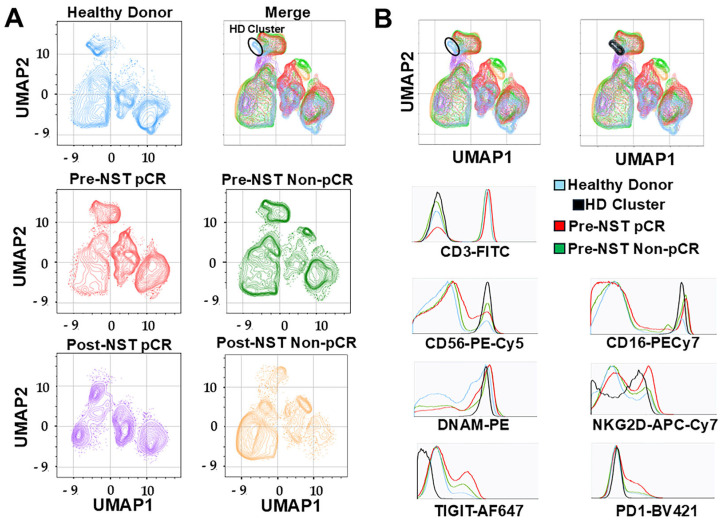
Multiparametric analysis to evaluate the expression of CD3, CD56, CD16, DNAM-1, NKG2D, TIGIT, and PD-1 was assessed on PBMCs from healthy donors (n = 35) and breast cancer patients (n = 34) stratified by pathological complete response (pCR) and non-pathological response (Non-pCR), both pre (NST) and post (NST) neoadjuvant systemic therapy. (**A**) UMAP dot plots showing clusters in different colors: Healthy Donors (blue), Pre-NST pCR (red), Pre-NST Non-PCR (green), Post-NST pCR (purple), and Post-NST Non-PCR (orange). This highlights the distinct differences among them. The study groups are shown to merge with the exclusive population of healthy donors, enclosed in a black circle. (**B**) UMAP from study groups shows a cluster named HD cluster (black) that is not present in all groups of breast cancer patients, the Pre-NST pCR group (red), and the Pre-NST Non-pCR group (green). Phenotype histograms show CD3, CD56, CD16, DNAM-1, NKG2D, TIGIT, and PD-1 expression patterns.

Next, we analyzed NK cell characteristics using high-parameter data analysis to assess the expression of activating and inhibitory receptors. Figure 3A shows a two-dimensional graphical representation of the distribution of peripheral blood mononuclear cells (PBMCs) after using the UMAP algorithm. The distributions in the HD and BC groups, stratified by neoadjuvant systemic therapy (NST) response, are shown in different colors (Figure 3A). Notably, we identified a unique cluster, predominantly present in the HD group (indicated by the black circle), that is markedly diminished or absent across all BC patient groups, regardless of treatment status or pathological response. This specific population, hereafter referred to as the “HD Cluster,” is isolated for comparative analysis of Pre-NST pCR and pre-NST Non-pCR breast cancer patients in Figure 3B.

To define the functional profile of the HD cluster, we analyzed the expression of lineage markers and key checkpoint molecules, including activating (NKG2D, DNAM-1) and inhibitory receptors (TIGIT, PD-1) (Supplementary Figure S2). As shown in the representative histograms in Figure 3B, the HD cluster consists of mature CD3^−^CD56^+^CD16^+^NK cells. Phenotypically, this population is characterized by a robust DNAM-1+NKG2D^+^-activating profile, coupled with negligible expression of the inhibitory receptors TIGIT and PD-1. In contrast, NK cell populations in BC patients before treatment demonstrated increased expression of inhibitory markers (TIGIT and PD-1). Collectively, these data indicate that the healthy immune landscape is defined by a specific NK cell cluster with high cytotoxic potential, which is systematically altered or lost in the setting of breast cancer and NST resistance (Supplementary Figure S3).

### 2.5. Differential Expression of NKG2D, DNAM-1, TIGIT, and PD-1 in NK Cells Based on NST Response

**Figure 4:**
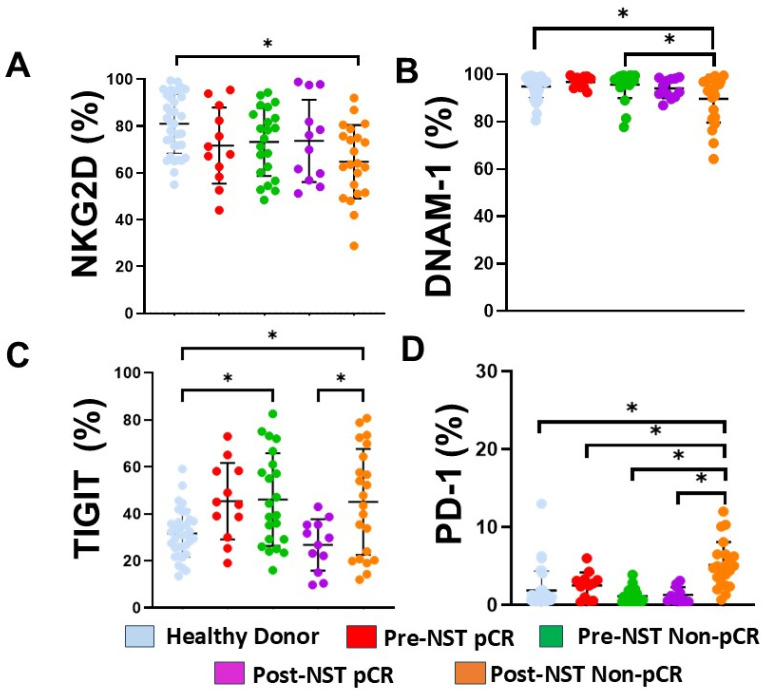
NKG2D, DNAM-1, TIGIT, and PD-1 expression were assessed on peripheral blood NK cells from healthy donors (n = 35) and breast cancer patients (n = 34), stratified by pathological complete response (pCR) and non-pathological response (Non-pCR), both pre- (pre-NST) and post- (post-NST) neoadjuvant systemic therapy. We evaluated the percentages of (**A**) NKG2D^+^ NK cells, (**B**) DNAM-1^+^ NK cells, (**C**) TIGIT^+^ NK cells, and (**D**) PD-1^+^ NK cells across the different groups. The Y-axis represents the frequencies of the different cell populations, while the X-axis displays the study groups, each depicted in a distinct color. Data are presented as individual percentages of expression along with their mean values. Statistical significance among the five groups was assessed using a one-way ANOVA, followed by Dunnett’s multiple-comparison test. Only statistically significant comparisons are shown: * *p* ≤ 0.05.

To characterize the functional phenotype of peripheral NK cells, we analyzed the expression of key activating and inhibitory receptors across all cohorts. Manual gating was employed to quantify the percentages of positive cells for each marker. Evaluation of NKG2D expression revealed a significant reduction in the frequency of NKG2D^+^NK cells in the Post-NST Non-pCR group compared to HD (Figure 4A, *p* < 0.05). For DNAM-1, although expression levels remained high across most groups, a significant decrease was observed in the post-NST Non-pCR cohort compared with both Healthy Donors and the pre-NST Non-pCR baseline (Figure 4B, *p* < 0.05).

The expression of inhibitory markers markedly differed according to clinical response. The proportion of TIGIT^+^ NK cells was significantly elevated in both pre- and post-NST Non-pCR patients compared with HD (Figure 4C, *p* < 0.05). Notably, following NST, TIGIT expression was significantly lower in the pCR group than in the Non-pCR group (*p* < 0.05), returning to levels comparable to those in HD. Regarding PD-1 expression (Figure 4D), levels were significantly higher in the post-NST Non-pCR cohort compared with the other groups, including Healthy Donors and post-NST pCR patients (*p* < 0.05).

Taken together, these results indicate that patients who did not achieve pCR have downregulation of activating receptors (DNAM-1, NKG2D) and significant upregulation of inhibitory checkpoints (TIGIT, PD-1). For CD3+CD56+ NKT-like cells, Non-responders showed clear increases in inhibitory TIGIT expression, while responder patients showed lower TIGIT expression (Supplementary Figure S4).

### 2.6. Post-Treatment Non-pCR Breast Cancer Patients Exhibit Higher Percentages of NK Cells Co-Expressing Inhibitory Receptors

**Figure 5:**
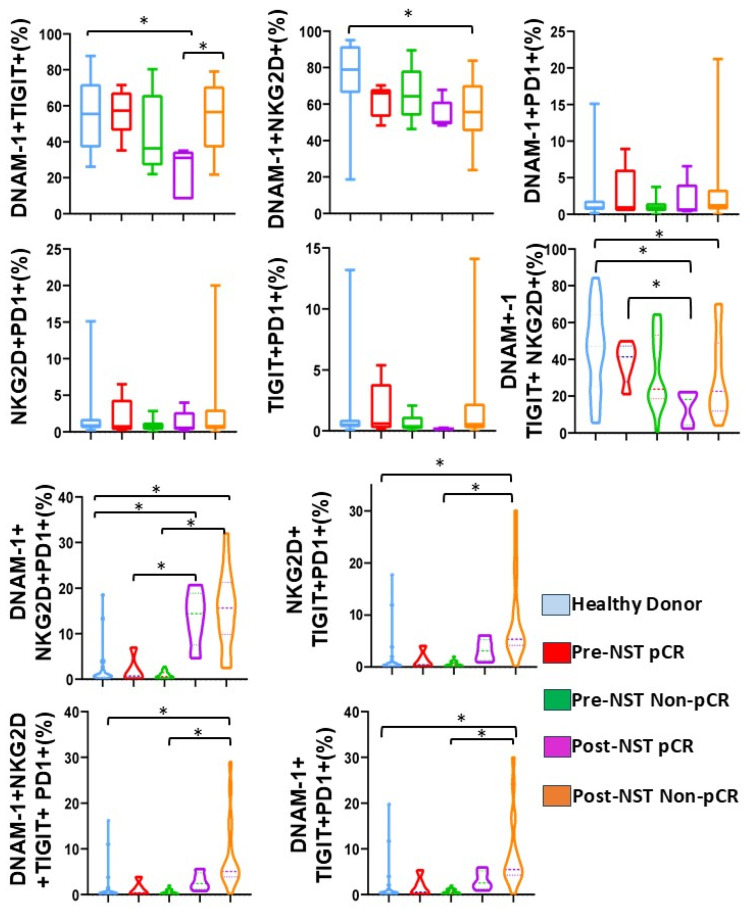
NKG2D, DNAM-1, TIGIT, and PD-1 co-expression was assessed on NK cells from healthy donors (n = 35) and breast cancer patients (n = 34), stratified by pathological complete response (pCR) and Non-pathological response (Non-pCR), both pre- (pre-NST) and post- (post-NST) neoadjuvant systemic therapy. We evaluated the percentages of DNAM-1^+^ TIGIT^+^ NK cells, DNAM-1+NKG2D+ NK cells, DNAM-1+PD-1+ NK cells, NKG2D+PD-1+ NK cells, TIGIT+PD-1+ NK cells, DNAM-1+TIGIT+NKG2D+ NK cells, DNAM-1+NKG2D+PD-1+, NKG2D+TIGIT+PD-1+ NK cells, DNAM-1+NKG2D+TIGIT+PD-1+, and DNAM-1+TIGIT+PD-1+NK cells, from the different groups. The Y-axis shows the frequencies of the other cell populations, and the X-axis displays the study groups, each in a distinct color. Data are presented as individual percentages of expression along with their mean values. Statistical significance among the five groups was assessed using a one-way ANOVA, followed by Dunnett’s multiple-comparison test. Only statistically significant comparisons are shown: * *p* ≤ 0.05.

We next assessed the co-expression patterns of activating (DNAM-1, NKG2D) and inhibitory (TIGIT, PD-1) receptors to determine the NK cell exhaustion receptor signature (Figure 5).

Analysis of co-expression showed that DNAM-1^+^TIGIT^+^ NK cells were significantly reduced in the post-NST pCR group compared to both HD and the post-NST Non-pCR group (*p* < 0.05). Conversely, the frequency of DNAM-1^+^ NKG2D^+^ NK cells was significantly lower in post-NST Non-pCR patients than in HD patients (*p* < 0.05). No significant differences were noted between DNAM-1^+^PD1^+^, NKG2D^+^PD-1^+^, or TIGIT^+^PD-1^+^ co-expression across the groups.

When analyzing the co-expression of DNAM-1^+^TIGIT^+^NKG2D^+^, we observed a reduction in their co-expression in the post-NST pCR patients compared with pre-NST pCR and HD (*p* < 0.05). Likewise, in Non-pCR patients, we observed a significant decrease in this co-expression compared with HD (*p* < 0.05). Furthermore, there was an increase in the co-expression of DNAM^+^NKG2D^+^PD-1^+^ in the post-NST pCR and post-NST Non-pCR groups compared to their pre-NST counterparts and HD (*p* < 0.05). Finally, analysis of receptor co-expression revealed that NKG2D^+^TIGIT^+^PD-1^+^, DNAM-1^+^TIGIT^+^PD-1^+^, and DNAM-1^+^NKG2D^+^TIGIT^+^PD-1^+^ NK cell subsets significantly increased in post-NST Non-pCR BC patients compared with the same patients at baseline and with Healthy Donors (*p* < 0.05).

In parallel, CD3^+^CD56^+^ NKT-like cells exhibited co-expression patterns similar to those observed in NK cells. Specifically, the frequencies of DNAM-1^+^PD-1^+^, DNAM-1^+^TIGIT^+^, NKG2D^+^TIGIT^+^PD-1^+^, DNAM-1^+^TIGIT^+^PD-1^+^, and DNAM-1^+^NKG2D^+^TIGIT^+^PD-1^+^ subsets decreased in pCR BC patients following NST (Supplementary Figures S5 and S6).

The accumulation of exhaustion signatures, like the NKG2D^+^TIGIT^+^PD-1^+^ and DNAM-1^+^NKG2D^+^TIGIT^+^PD-1^+^, identifies a state of NK cell dysfunction in Non-pCR breast cancer patients post-NST. These findings establish that the systemic co-expression of multiple checkpoints in both NK and CD3^+^CD56^+^ NKT-like cells serves as a biomarker of therapeutic resistance and impaired NK cells in breast cancer.

### 2.7. Peripheral Blood NK Cells from Breast Cancer Patients with pCR Showed Increased Cytotoxicity After Therapy

**Figure 6:**
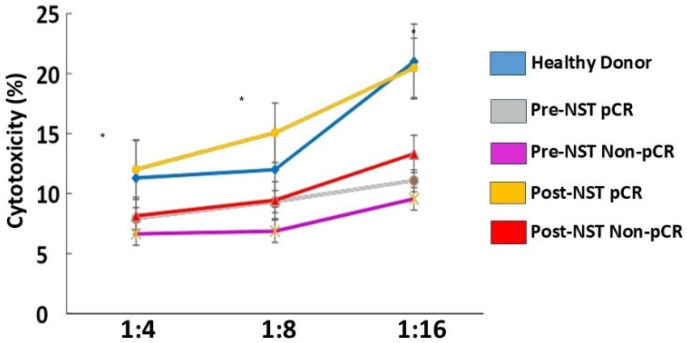
Evaluation of NK cell cytotoxicity was assessed in PBMC samples from healthy donors (n = 35) and breast cancer patients (n = 34), stratified by pathological complete response (pCR) and Non-pathological response (Non-pCR) for both pre- (pre-NST) and post- (post-NST) neoadjuvant systemic therapy. PBMC NK cells were co-cultured with target cells (K562). The co-culture was performed at different ratios (1:4, 1:8, and 1:16) with the target cell (K562) and effector cells (PBMC NK cells). The cytotoxic effect of peripheral blood NK cells against K562 cells was evaluated in Healthy Donors and in pretreatment and post-treatment breast cancer patients, with or without pathological response. The Y-axis represents NK cell cytotoxic activity, while the X-axis shows the study groups, each depicted in a distinct color. Data are presented as individual percentages of expression along with their mean values. Statistical significance among the five groups was assessed using a one-way ANOVA, followed by Dunnett’s multiple-comparison test. Only statistically significant comparisons are shown: * *p* ≤ 0.05.

We evaluate NK cell-mediated cytotoxicity in K562 cells across the different study groups. Post-NST pCR breast cancer patients exhibited NK cell activity comparable to that of Healthy Donors. Post-NST pCR breast cancer patients showed an increase in NK cell cytotoxicity compared with pretreatment levels in Pre-NST pCR patients or with Non-pCR breast cancer patients before or after NST, *p* ≤ 0.05 (Figure 6).

The restoration of NK cell cytotoxic activity to Healthy-Donor levels in pCR breast cancer patients after NST demonstrates a functional recovery of the NK cell response.

### 2.8. IL-2, sFASL, and Granzyme B Are Increased in Breast Cancer Patients with pCR After Treatment. Meanwhile, IL-2, IL-10, TNF-α, sFASL, and Granzyme B Are Decreased in Non-pCr Patients After Treatment

**Figure 7:**
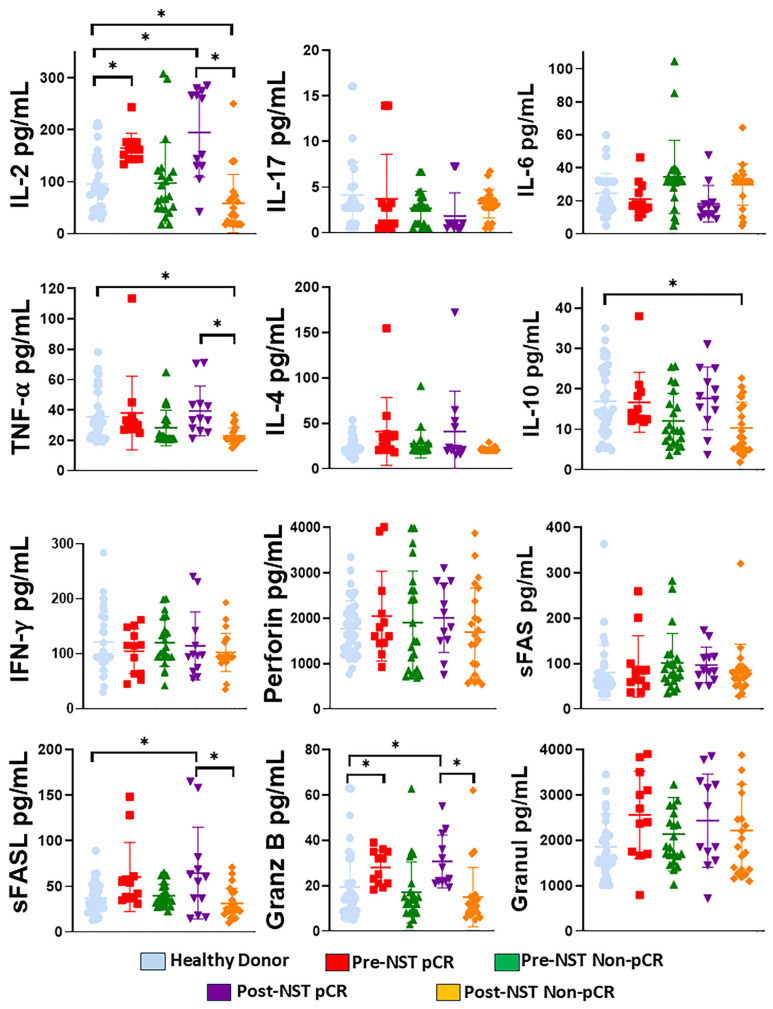
Evaluation of cytokines related to NK cell activity (IL-2, IL-17A, IL-6, TNF-α, IL-4, IL-10, and IFN-γ), sFAS, sFAS-L perforin, granzyme B, and granulysin in the serum of healthy donors (n = 35) and breast cancer patients (n = 34), stratified by pathological complete response (pCR) and Non-pathological response (Non-pCR), for both pre- (pre-NST) and post- (post-NST) neoadjuvant therapy. Serum levels of IL-2, IL-17A, IL-6, TNF-α, sFAS, sFAS-L, IL-4, IL-10, IFN-γ, Perforin, Granzyme, and granulysin (pg/mL) were measured across the groups. The Y-axis represents concentrations in pg/mL, and the X-axis displays the study groups, each in a distinct color. Data are presented as individual concentrations, with their means. Statistical significance among the five groups was assessed using a one-way ANOVA, followed by Dunnett’s multiple-comparison test. Only statistically significant comparisons are shown: * *p* ≤ 0.05. Granz B (Granzyme B), Ganul (Granulysin).

Serum NK-associated cytokines were compared between HD and BC patients, as well as before and after NST (Figure 7). Post-NST Non-PCR patients exhibited lower IL-2, TNF-α, sFASL, and Granzyme B concentrations than post-NST pCR patients (*p* < 0.05). Similarly, post-NST Non-pCR patients showed lower IL-2, TNF-α, and IL-10 levels than HD (*p* < 0.05). Finally, we observed increased IL-2, sFASL, and Granzyme B levels in post-NST pCR patients compared with Healthy Donors (*p* < 0.05). Likewise, pre-NST pCR patients have higher concentrations of IL-2 and Granzyme B than Healthy Donors (*p* < 0.05). No significant differences were observed in the concentrations of IL-17A, IL-6, IL-4, IFN-γ, Perforin, sFAS, or granulysin among the groups. Altogether, these results indicate that the balance and regulation of cytokines such as IL-2, TNF-α, and IL-10 are important for immune responses against breast cancer. Regulation of granzyme B and sFASL is important for NK cell and CD8+ T cell activity against breast cancer cells.

The decrease in IL-2, TNF-α, sFASL, and Granzyme B in Non-pCR breast cancer patients following therapy indicates impaired effector capacity of their NK cells. Likewise, the increase in IL-2, sFASL, and Granzyme B in pCR patients indicates restoration of NK cell cytotoxicity, as observed in the cytotoxicity assay.

## 3. Discussion

In this study, we evaluated the clinical and immunological landscapes of BC patients undergoing NST, a critical intervention given that BC remains a leading cause of global cancer mortality [1]. We observed that 23.8% of patients achieved pCR, a rate consistent with those reported in established neoadjuvant clinical trials and larger molecular subtype studies [5,29,30]. Despite advances in staging and molecular classification [31,32], reliable, non-invasive biomarkers for pCR remain an unmet clinical need [3]. NK cells are essential mediators of anti-tumor immunity through their innate cytotoxic capabilities [10,31].

Our findings revealed that CD56^bright^ NK cells were elevated after neoadjuvant therapy in Non-PCR patients, while the CD56^dim^ subset was significantly decreased. The reduction in the cytotoxic CD56^dim^ population, which represents the primary effector subset, suggests that NK cell-mediated anti-tumor immunity is inherently compromised in breast cancer patients with Non-pCR. CD56^bright^ NK cells primarily have a regulatory role through cytokine production rather than direct lysis [32]. This imbalance in NK cell proportions was functionally validated by our cytotoxicity assays, which demonstrated a marked reduction in lytic activity in Non-pCR patients post-treatment compared to those who achieved pCR.

These observations align with and extend recent investigations into the role of NK cells in breast cancer immunosurveillance. It has been reported that, although total peripheral NK cell percentages do not differ significantly across clinical stages, intrinsic subtypes, or chemotherapy regimens, the functional quality of these cells remains a critical variable [33]. We observed changes in total NK cell numbers among Non-pCR breast cancer patients and a profound shift in NK cell subset distribution.

Furthermore, the decrease in the CD56^dim^ population observed in our Non-pCR cohort after NST is consistent with findings in other studies [34,35]. Their characterization of NK cell phenotypes in breast cancer patients also revealed systemic immune suppression, specifically through lower expression of activating markers, such as NKG2D and CXCR3 (C-X-C motif chemokine receptor 3), across both CD56^bright^ and CD56^dim^ subsets compared to HD. Additionally, they highlighted that suppression of these compartments is often associated with upregulation of inhibitory pathways, such as PD-1 and TGF-β RII, further corroborating our observation of an exhausted immune landscape in therapy-resistant patients [34].

Peripheral blood NK cells can be characterized not only by the expression of CD56, an adhesion molecule mediating homotypic adhesion, but also by CD16 (FcRγIII). In this study, we analyzed four NK cell subpopulations defined by CD56 and CD16 expressions, as these subsets represent distinct functional or maturation states and their distribution has been linked to various diseases [36]. The populations were categorized as CD56^bright^CD16^neg^ NK cells, CD56^bright^CD16^pos^ NK cells, CD56^dim^CD16^neg^ NK cells, and CD56^dim^CD16^pos^ NK cells. Our results demonstrate that the CD56^bright^CD16^neg^ and transitional CD56^bright^CD16^pos^ subsets significantly expanded in the post-NST Non-pCR group. In contrast, the terminal effector population, CD56^dim^CD16^pos^, which possesses the highest cytotoxic capacity, further declined following therapy. CD56^dim^CD16^neg^ NK cells increased post-treatment specifically in the pCR group; this phenomenon is consistent with activation-induced metalloprotease-mediated cleavage of CD16, suggesting heightened NK activation in responders [37,38].

High-parameter UMAP analysis corroborated these findings, identifying a unique CD3^−^CD56^+^CD16^+^ cluster in HD characterized by a high density of activating receptors (DNAM-1+, NKG2D+) and negligible levels of inhibitory markers (TIGIT^neg^, PD-1^neg^), which was systematically lost in BC patients.

Contact-dependent communication between the TME and cancer cells is partly mediated by paracrine signaling. Cancer cells overexpress the immune checkpoint PD-L1, which engages the PD-1 receptor on Natural Killer cells, thereby suppressing immune surveillance. NK cells also express immune checkpoint receptors such as PD-1 and TIGIT, which are well-studied in T cell activation and can also inhibit NK cell activation. In cancer, tumors can highly express the ligands of these molecules, using this mechanism to inhibit T- or NK-cell activation and promote resistance to immune checkpoint blockade therapies [39,40,41].

In our study, we assessed the expression of two checkpoint receptors (TIGIT and PD-1) and two activating receptors (DNAM-1 and NKG2D) on NK cells. We observed a striking imbalance in the TIGIT/DNAM-1 axis: TIGIT was significantly elevated in BC patients but decreased post-NST primarily in the pCR group, while DNAM-1, which competes for the same ligands (CD112 and CD155), was reduced in Non-pCR breast cancer patients. Furthermore, Non-pCR patients demonstrated a significant increase in PD-1 expression and complex co-expression patterns, such as NKG2D^+^TIGIT^+^PD-1^+^ and DNAM-1^+^NKG2D^+^TIGIT^+^PD-1^+^, following NST. These phenotypic shifts directly correlated with our functional assays. Cytotoxicity assays revealed reduced activity in Non-pCR patients after treatment compared to those who achieved pCR. While cytotoxicity in pCR patients trended toward HD levels, Non-pCR patients remained significantly impaired.

The soluble molecule profiles in the serum of Non-pCR breast cancer patients indicate failure of effector immune functions. This aligns with our observations in NK cell populations and with the expression of TIGIT and PD-1 receptors in these patients. The reduction of IL-2, TNF-α, sFASL, and granzyme B after therapy in Non-pCR breast cancer patients suggests that NK cells in the peripheral immune compartment remain dysfunctional. Prior studies show that immune checkpoint expression on NK cells is associated with reduced IFN-γ and granzyme B production [42,43].

After treatment, we observed a reduction in sFASL and Granzyme B in the Non-pCR breast cancer patients, which directly correlates with the decrease in NK cell cytotoxicity against K562 cells observed in our study. As NK cell-mediated cytotoxicity depends on the availability of lytic granules and FasL signaling [44], lower serum levels of these molecules indicate reduced systemic NK cell activity in these patients.

The reduced levels of IL-2 and TNF-α in patients with Non-pCR breast cancer highlight the importance of cytokines and their regulation; in particular, decreased IL-2 has been shown to promote tumor progression and escape and may represent promising therapeutic targets [45,46].

Many studies focus only on single-point measurements of soluble molecules. Our longitudinal analysis allowed us to track changes in cytokines and molecules, including Granzyme and FasL, before and after therapy. Our study suggests that NST does not reverse NK cell dysfunction in patients who did not achieve pCR.

Collectively, these findings suggest that breast cancer patients who achieve a pathological complete response (pCR) have a systemic recovery of the NK cell compartment and its function. In contrast, Non-pCR breast cancer patients show dysfunctional NK cells, characterized by decreased mature cytotoxic CD56^dim^ cells and increased immature CD56^bright^ cells. This alteration is further associated with imbalances in the TIGIT/DNAM-1 axis and the emergence of co-expression patterns that more closely link to suppression of the NK cell-mediated response. Supporting these observations, Non-pCR breast cancer patients exhibit decreased NK cell cytotoxicity and reduced peripheral serum levels of granzyme B, sFASL, and IL-2.

While this study provides evidence on NK cell subpopulations and the expression of activating and inhibitory receptors, the inherent molecular heterogeneity of breast cancer subtypes suggests that future multicenter studies with larger, stratified cohorts could validate our findings and emphasize the potential of NK cell function as a biomarker of treatment response. Another limitation of this study is that we analyze only changes in peripheral blood NK cells. Future studies correlating peripheral blood NK cells with tumor-infiltrating lymphocytes (TILs) could offer additional insights into cellular trafficking patterns toward the tumor microenvironment. Furthermore, by prioritizing the longitudinal analysis of peripheral blood, this research establishes a foundational framework for developing minimally invasive, clinically accessible biomarkers.

## 4. Materials and Methods

A prospective cohort study was conducted at Hospital de Especialidades, Centro Médico Nacional de Occidente Ignacio García Tellez, a tertiary care hospital in Western Mexico, from October 2022 to October 2023. Patients were eligible for inclusion in the BC group if they: (a) were newly diagnosed with primary BC; (b) had an Eastern Cooperative Oncology Group performance status (ECOG-PS) of 0–2 [11]; and (c) presented with locally advanced disease (stage II or III) according to the American Joint Committee on Cancer (AJCC) 8th edition [12]. All eligible patients were candidates for neoadjuvant systemic therapy (NST) and provided written informed consent.

Patients with previous active cancer therapy, active cardiac disease, and pregnancy were excluded. A control group of healthy donors was also included; these patients underwent routine breast screening (mammography with BI-RADS 1 or 2 and were age-matched ± 5 years to the BC group).

Baseline characteristics were collected through medical evaluation and peripheral blood samples. A trained researcher reviewed medical records to obtain baseline data, including clinical variables such as comorbidities and tumor characteristics.

Menopause was defined as the absence of menstruation for at least 1 year [13]. A sedentary lifestyle was defined as the absence of at least 30 min of physical activity per day for 4 days a week. Body Mass Index (BMI) was estimated as weight (kg)/height (m) and categorized as low weight (<18.5 kg/m^2^), normal weight (18.5 to 24.9 kg/m^2^), overweight (25 to 29.9 kg/m^2^), and obesity (≥30 kg/m^2^) [14].

Peripheral blood samples were collected from all groups using Vacutainer tubes with EDTA anticoagulant (BD Biosciences, San Jose, CA, USA) for immunophenotypic analysis by flow cytometry. Heparin anticoagulant tubes were used to collect samples (BD Biosciences, San Jose, CA, USA) for the cytotoxicity assay. For the BC group, basal samples were collected 1 week before the first cycle of neoadjuvant systemic therapy, and the final sample was collected 1 week before surgical resection. In the healthy donor group, the blood samples were collected after a breast cancer screening mammogram.

Tumor phenotype was classified based on immunohistochemical assessment of hormone receptor expression (estrogen receptor [ER] and progesterone receptor [PR]), Ki-67 labeling index, and human epidermal growth factor receptor 2 (HER2) overexpression [47]. The physician selected neoadjuvant therapy with anthracyclines and taxanes, trastuzumab, and pertuzumab, in accordance with established guidelines and multidisciplinary discussions. Histological grading was performed using the Scarff Bloom Richardson classification, which was classified as grade 1 (well differentiated), grade 2 (moderately differentiated), or grade 3 (poorly differentiated) [48].

### 4.1. Outcome Measure: Pathological Complete Response

Tumor response was analyzed in the excised surgical specimens, and pathological complete response (pCR) was defined as the absence of invasive tumor in the surgical specimens and the axillary lymph nodes (ypT0/ypTis and ypN0) based on histopathological analysis. Residual tumors were classified as a non-pathological response (Non-pCR) [30].

### 4.2. PBMC Isolation

A density gradient was prepared from whole blood using Histopaque-1077 (Sigma-Aldrich, St Louis, MO, USA), and peripheral blood mononuclear cells (PBMCs) were isolated. Viability was determined by trypan blue staining, which was greater than 95%. The PBMCs were stained for flow cytometry analysis.

### 4.3. PBMC Staining by Flow Cytometry Analysis

PBMCs (1 × 10^6^) were stained to identify CD56^dim^ and CD56^bright^ NK cell populations and the expression of PD-1, TIGIT, NKG2D, and DNAM-1. For this, PBMCs were stained with CD56-PECy5, CD16-PE-Cy7, CD3-FITC, PD-1-BV421, TIGIT-Alexa Fluor 647, NKG2D-APC-Cy7, and DNAM-1-PE antibodies (Biolegend, San Diego, CA, USA). Samples were incubated for 30 min at room temperature, protected from light. After that, 2 mL of PBS (buffer solution, Sigma-Aldrich, St Louis, MO, USA) was added. Cells were centrifuged at 1800 rpm for 7 min, decanted, and 500 µL of PBS was added. Samples were analyzed on a CYTOFLEX flow cytometer (Beckman Coulter, Brea, CA, USA). At least 300,000 events were acquired from the lymphocyte region. Expression of NK cell populations and their receptors was analyzed using FlowJo v10.0 (BD Biosciences, Ashland, OR, USA). To determine positivity, fluorescence-minus-one controls were used, and during analysis, the CD3-CD56- cell population was used as an internal control for gating. High-parameter data analysis of the lymphocyte population was performed on concatenated samples using FlowJo v10.0. First, quality control was performed using PeacoQC (Peak-based selection of high-quality cytometry data) [49]. We then gated on single events and the lymphocyte region. We used UMAP (Uniform Manifold Approximation and Projection) for dimension reduction [50]. Phenograph was used to define the different clusters, while Cluster Explorer was used to evaluate their characteristics [51].

### 4.4. PBMC NK Cell Cytotoxicity Assay

Cell counting was performed, and PBMC NK cells were co-cultured with target cells (K562, CCL-243 ATCC, Manassas, VA, USA); the co-culture was performed in different proportions (1:4, 1:8, and 1:16), with one target cell (K562) for 4, 8, or 16 effector cells (PBMC NK cells). Thirty thousand target cells were used as a fixed range. The cells were incubated in RPMI medium (200 µL) for 18 h. Subsequently, we collected the supernatant from the co-cultured cells. We measured LDH (Lactate Dehydrogenase) activity using the CyQUANT LDH Cytotoxicity Assay Kit (Thermo Fisher, Waltham, MA, USA) according to the manufacturer’s instructions. Then, the LDH absorbance was measured using a microplate reader at 490 nm and 680 nm (Sinergy HT Multi-Mode Microplate Reader, Bio Tek, Winooski, VT, USA). At the end of incubation, 25 μL of each sample was transferred to a 96-well flat-bottom plate, and 25 μL of the reaction mixture was added to each well. To calculate LDH activity, we subtracted the absorbance at 680 nm from that at 490 nm. To estimate cytotoxicity percentages, the following equation was applied to the corrected values: % cytotoxicity = (Experimental value − Effector cells spontaneous control − Target cells spontaneous control)/(Target cells maximum control − Target cells spontaneous control) × 100.

### 4.5. Determination of IL-17A, IL-2, IL-4, IL-10, IL-6, TNF-α, Fas, FasL, IFN-γ, Granzyme A, Granzyme B, Perforin, and Granulysin

A Human Legendplex kit from BioLegend (San Diego, CA, USA) was used to evaluate *IL-17A, IL-2, IL-4, IL-10, IL-6, TNF-α, Fas, FasL, IFN-γ, Granzyme A, Granzyme B, Perforin, and Granulysin*. For this, we added 25 μL of the assay buffer, 25 μL of standard, or 25 μL of serum sample to the designated tubes. Subsequently, 25 μL of beads coated with specific antibodies for the cytokines to be determined were added, and samples were incubated for 2 h (shaking, protected from light, at room temperature). Then, the samples were washed, and 25 μL of the detection antibodies were added. Samples were incubated for 1 h. After incubation, 25 μL of streptavidin-PE was added to the samples and incubated for 30 min (at room temperature, protected from light under shaking). Washing was performed using assay buffer, and the samples were resuspended in 200 μL of wash buffer. The samples were acquired on the CYTOFLEX flow cytometer (Beckman Coulter, Brea, CA, USA), (300 events from each bead were acquired), and data were analyzed using LegendPlex software v8. Data represent the mean of the concentration (pg/mL) ± standard deviation of each cytokine.

### 4.6. Ethics

The research and ethics committee of the Hospital de Especialidades Centro Médico Nacional de Occidente approved informed consent for sample collection and follow-up (approval number R-2022-1303-206, October 2022). This study was conducted in accordance with the International Conference on Harmonization Guidelines, Good Clinical Practice, and Declaration of Helsinki.

### 4.7. Statistical Analysis

Quantitative variables were expressed as means ± standard deviations (SDs), and qualitative variables were expressed as frequencies and percentages (%). The chi-square test or Fisher’s exact test was performed to compare qualitative variables. One-way ANOVA, followed by Dunnett’s post hoc test, was conducted to compare the five groups: (a) Healthy Donor group, (b) pre-NST with pCR, (c) pre-NST with Non-pCR, (d) post-NST with pCR and (e) post-NST with Non-pCR. Statistical significance was set at *p* < 0.05. Software Graphpad v10.3.1 (LLC d.b.a. Dotmatics, San Diego, CA, USA). To ensure the statistical validity of comparing groups with unequal sample sizes (n = 12 and n = 22) in breast cancer patients with pCR or Non-pCR, a post hoc power analysis was conducted using G*Power (v3.1.9.7, University of Düsseldorf, Germany).

## 5. Conclusions

Patients with breast cancer who achieve pathological complete response (pCR) following neoadjuvant systemic therapy (NST) have systemic restoration of the TIGIT/DNAM-1 axis and substantial recovery of natural killer (NK) cell-mediated cytotoxicity, accompanied by elevated levels of interleukin-2 (IL-2), soluble FAS ligand (sFASL), and granzyme B. Likewise, NK cells in Non-pCR patients remain functionally impaired. Our study demonstrates that achieving a pathological complete response in patients with breast cancer is associated with significant improvements in NK cell subsets and their functional capacity.

## Figures and Tables

**Table 1:** Comparison of clinical variables of the breast cancer group with pathological complete response versus non-pathological complete response.

Variable	pCR n = 12	Non-pCR n = 22	*p*-Value
Age (years)	52.8 ± 14.1	56.5 ± 14.3	0.47
Contraceptive use, n (%)	3 (25)	11 (50)	0.27
Clinical stage, n (%)			0.1
IIA	2 (17)	6 (27)	
IIB	3 (25)	5 (23)	
IIIA	2 (17)	9 (41)	
IIIB	4 (33)	2 (9)	
IIIC	1 (8)	0	
Tumor phenotype, n (%)			0.03
ER+/PR+	0	5 (23)	
ER+/PR−	2 (16)	9 (41)	
HER2 +++	5 (42)	6 (27)	
Triple negative	5 (42)	2 (9)	
Histological grade, n (%)			0.13
Well-differentiated	2 (17)	1 (4)	
Moderately differentiated	6 (50)	16 (72)	
Poorly differentiated	4 (33)	4 (18)	

Age is expressed as mean ± standard deviation. The data are expressed as the number of cases (n) and frequencies (%). ER (Estrogen Receptor), PR (Progesterone Receptor), HER2 (Human Epidermal Growth Factor Receptor 2).

## Data Availability

The original contributions presented in this study are included in the article/Supplementary Material. Further inquiries can be directed to the corresponding authors.

## Supplementary Materials

The following supporting information can be downloaded at https://www.mdpi.com/article/10.3390/ijms27041634/s1.

## Abbreviations

The following abbreviations are used in this manuscript:

BC	Breast cancer
HD	Healthy donors
NK cells	Natural killer cells
pCR	Pathological complete response
Non-pCR	No pathological complete response.
DNAM-1	DNAX accessory molecule-1
NKG2D	Natural killer group 2, member D
PD-1	Programmed cell death protein 1
TIGIT	T cell immunoreceptor with Ig and ITIM domains
IL-2	Interleukin-2
TNF-α	Tumor necrosis factor-alpha
IFN-γ	Interferon-gamma
IL-10	Interleukin-10
TME	Tumor microenvironment
PBMCs	Peripheral blood mononuclear cells
CXCR3	C-X-C motif chemokine receptor 3
BMI	Body mass index
