# Supplementary material for: Natural Killer Cell Phenotype and Function as a Predictive Factor for Treatment Response to Neoadjuvant Therapy in Breast Cancer Patients
Source: Int J Mol Sci. 2026 Feb 7;27(4):1634. doi: 10.3390/ijms27041634 (PMC12940497; doi:10.3390/ijms27041634)
Supplement: Supplementary file 1 [file ijms-27-01634-s001.zip › ijms-4117619-supplementary.pdf]

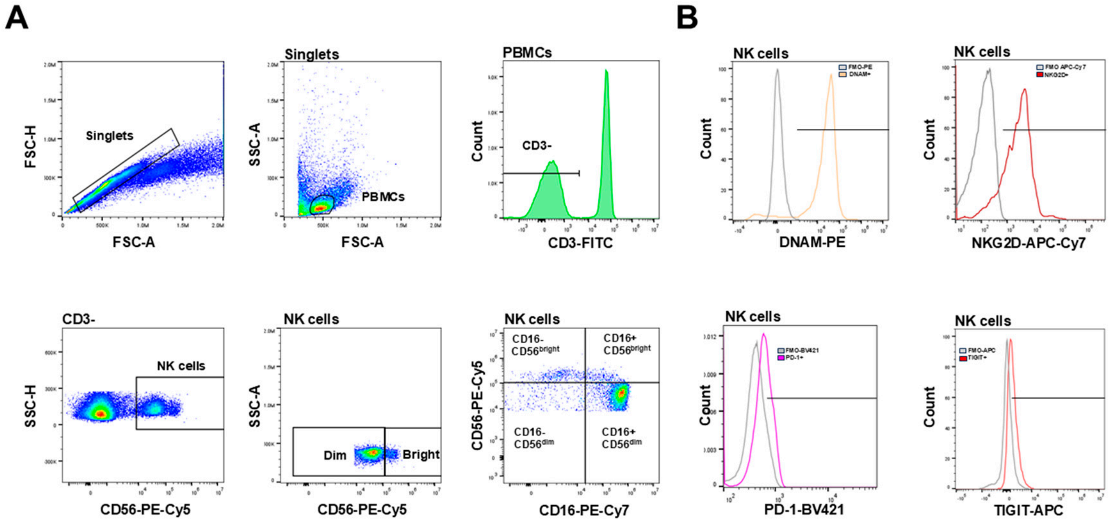

Supplementary Figure S1. Gating strategy used to identify natural killer (NK) cell populations and the expression of NKG2D, DNAM-1, TIGIT, and PD-1 receptors. (A) We first gated on singlets, then selected the PBMC region, and finally selected CD3- cells. Subsequently, we gated on CD56+ CD3- cells to precisely identify NK cells. We then use the CD56 pattern to distinguish between CD56 bright and CD56 dim NK cells. Further, the expression pattern of CD56 and CD16 helped us identify four populations of NK cells ($CD56^{dim}$CD16+, $CD56^{dim}$/CD16-, $CD56^{bright}$/CD16+, $CD56^{bright}$/CD16-). (B) From the NK cell population, we analyzed the expression and co-expression of the NKG2D, DNAM-1, TIGIT, and PD-1 receptors.

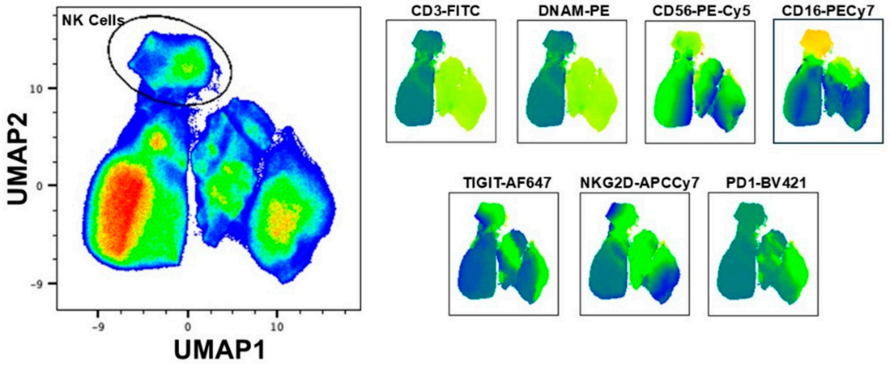

Supplementary Figure S2. UMAP visualization of PBMC NK cells from healthy donors and breast cancer patients. The figure shows CD3, DNAM-1, CD56, CD16, TIGIT, NKG2D, PD-1 expression pattern.

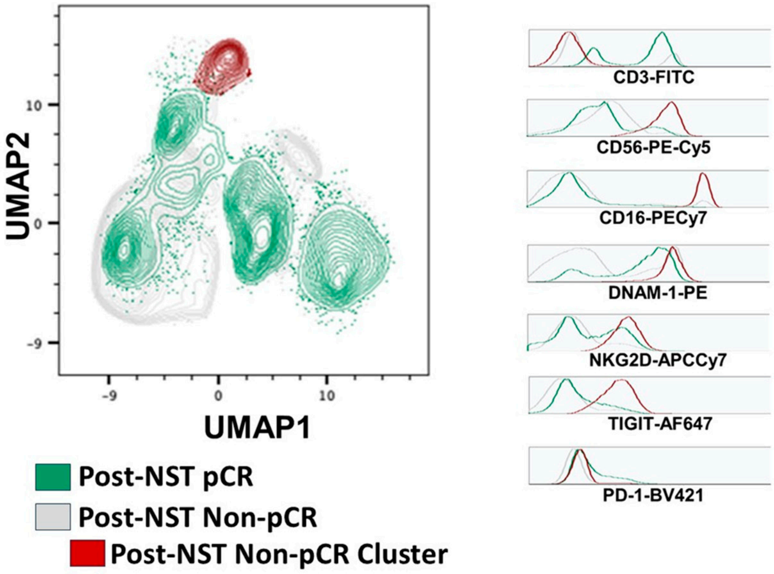

Supplementary Figure S3. The UMAP shows distinct clusters among post-NST breast cancer patients with (pCR, green) and without (Non-pCR, gray) pathological complete response, based on expression of CD3, DNAM-1, CD56, CD16, TIGIT, NKG2D, PD-1. The UMAP representation particularly reveals a cluster of non-pathological responses in breast cancer patients after treatment (brown). The histograms illustrate the expression of the different receptors.

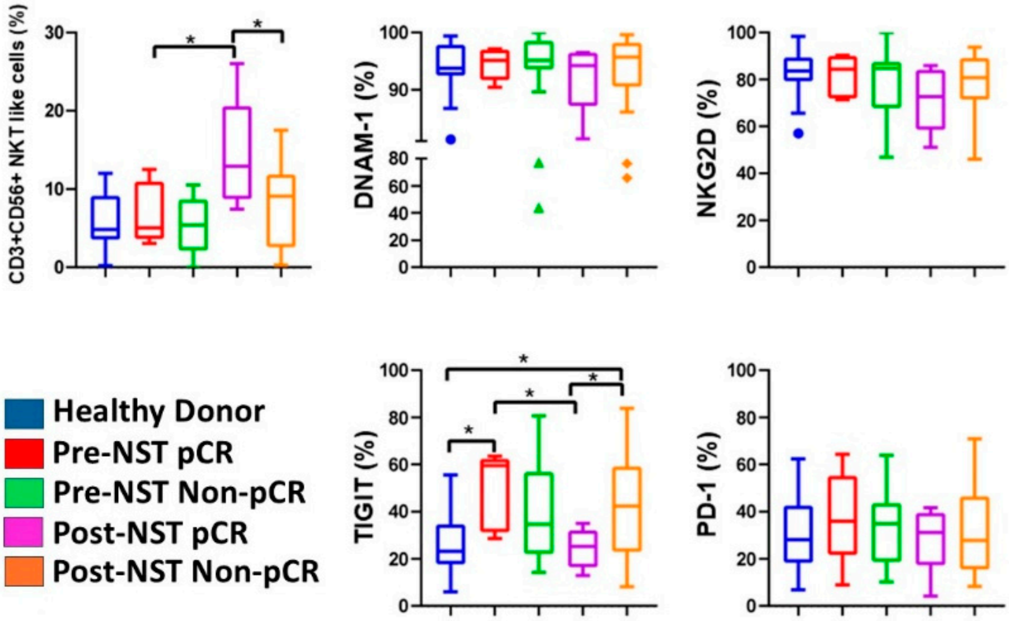

Supplementary Figure S4. Expression of NKG2D, DNAM-1, PD-1, and TIGIT in NKT-like cells from healthy donors (n=35) and breast cancer patients (n=34) with pathological complete response (pCR) and non-pathological response (Non-pCR) before (pre-NST) and after (post-NST) treatment. We evaluated the percentages of CD3+CD56+ NKT-like cells, DNAM-1+ NKT-like cells, NKG2D+ NKT-like cells, TIGIT+ NKT-like cells, and PD-1+ NKT-like cells

from the different groups. One-way ANOVA, followed by Dunnett's post-hoc test, was conducted to compare the five groups. A $p < 0.05$ was considered statistically significant between groups. Significant comparisons are displayed in the figures. The analysis for CD3+CD56+NKT-like cells was performed similarly to that of NK cells; after singlet cells, a graph between CD3 vs CD56 was performed, and the double positive population (CD3+CD56+) was analyzed for the pattern of expression and co-expression of DNAM-1, NKG2D, TIGIT, and PD-1.

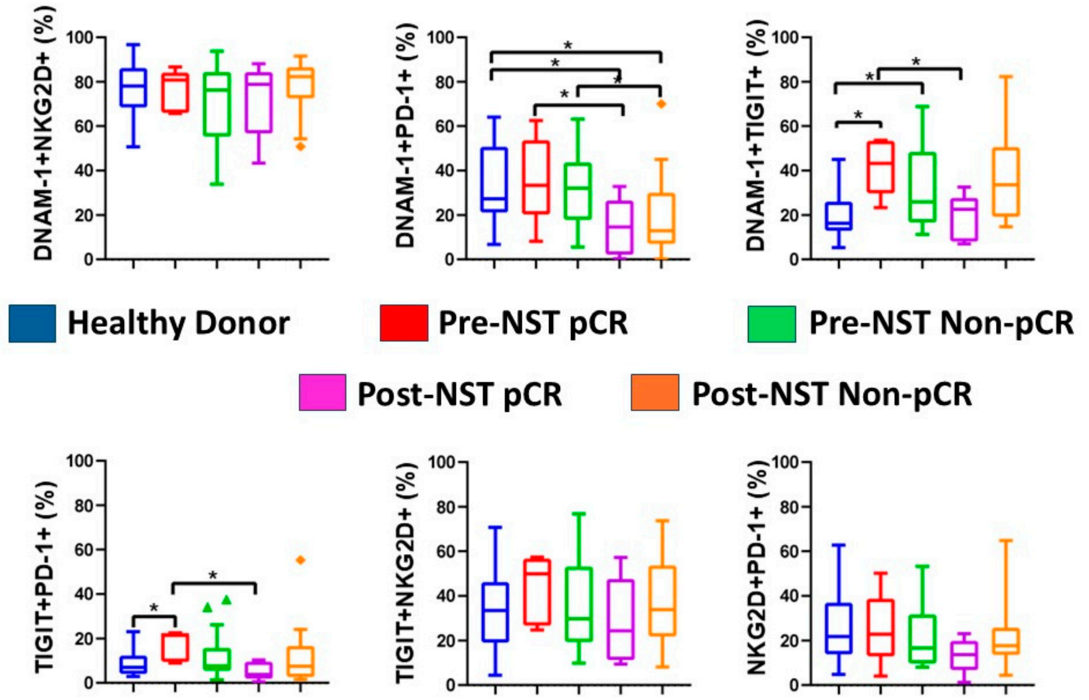

Supplementary Figure S5. Co-expression of NKG2D, DNAM-1, PD-1 and TIGIT in NK cells from healthy donors (n=35) and breast cancer patients (n=34) with pathological complete response (pCR) and non-pathological response (Non-pCR) before (pre-NST) and after (post-NST) treatment. We evaluated the percentages of DNAM-1+NKG2D+ NKT-like cells, DNAM-1+PD-1+ NKT-like cells, DNAM1+TIGIT+ NKT-like cells, TIGIT+PD-1+ NKT-like cells, TIGIT+NKG2D+ NKT-like cells, and NKG2D+PD-1+ NKT-like cells from different groups. One-way ANOVA, followed by Dunnett's post-hoc test, was conducted to compare the five groups. A $p < 0.05$ was considered statistically significant between groups. Significant comparisons are displayed in the figures.

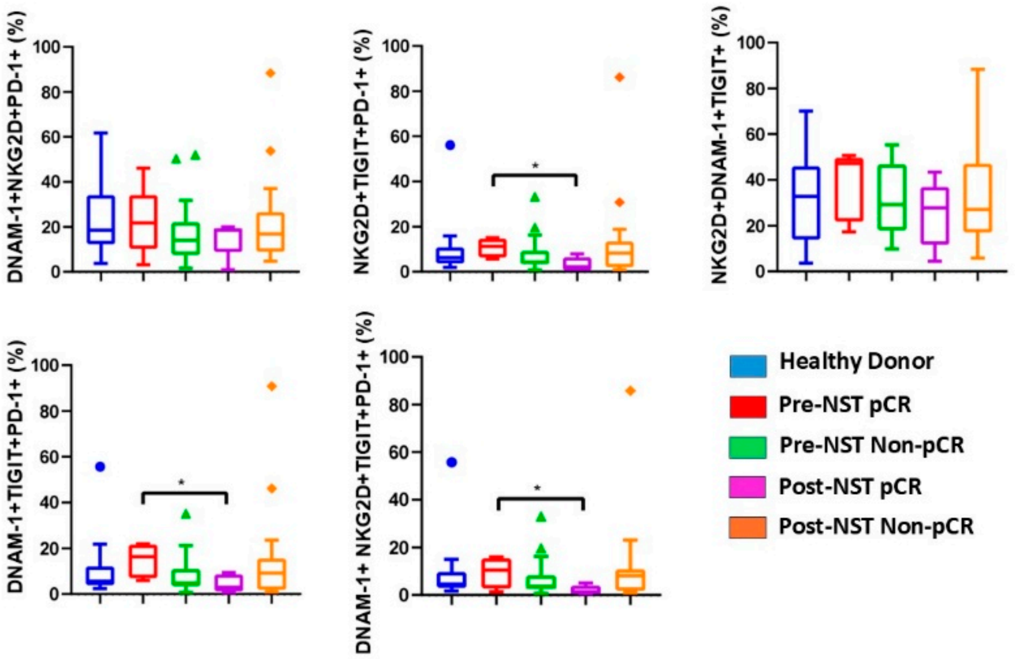

Supplementary Figure S6. Co-expression of NKG2D, DNAM-1, PD-1, and TIGIT in NK cells from healthy donors (n=35) and breast cancer patients (n=34) with pathological complete response (pCR) and non-pathological response (Non-pCR) before (pre-NST) and after (post-NST) treatment. We evaluated the percentages of DNAM-1+NKG2D+ PD-1+NKT-like cells, NKG2D+TIGIT+PD-1+ NKT-like cells, NKG2D+DNAM-1+TIGIT+ NKT-like cells, DNAM-1+TIGIT+PD-1+ NKT-like cells, and DNAM-1+NKG2D+TIGIT+PD-1+ NKT-like cells from different groups. One-way ANOVA, followed by Dunnett's post-hoc test, was conducted to compare the five groups. A $p < 0.05$ was considered statistically significant between groups. Significant comparisons are displayed in the figures.
